# NEO6860, modality-selective TRPV1 antagonist: a randomized, controlled, proof-of-concept trial in patients with osteoarthritis knee pain

**DOI:** 10.1097/PR9.0000000000000696

**Published:** 2018-10-26

**Authors:** Pierre Arsenault, Dan Chiche, William Brown, Jeffrey Miller, Roi Treister, Richard Leff, Philippe Walker, Nathaniel Katz

**Affiliations:** a Diex Recherche Sherbrooke, Sherbrooke, QC, Canada; b NEOMED Institute, Montreal, QC, Canada; c Consultant Exploratory Medicines, Vancouver, BC, Canada; d Faculty of Social Welfare and Health Sciences, The Cheryl Spencer Department of Nursing, University of Haifa, Haifa, Israel; e LLC, Chadds Ford, PA, USA; f Analgesic Solutions, Natick, MA, USA; g Department of Anaesthesiology and Perioperative Medicine, Tufts University School of Medicine, Boston, MA, USA

**Keywords:** NEO6860, TRPV1 antagonist, Osteoarthritis of the knee, Analgesic effect, Hyperthermia, Change in heat pain perception, Staircase test

## Abstract

**Introduction:**

NEO6860 is a TRPV1 antagonist when activated by capsaicin but not by heat or pH, developed to relieve pain without the adverse events reported with non–modality-selective TRPV1 antagonists.

**Objective:**

The primary Objective of this study was to evaluate the analgesic efficacy and safety of NEO6860 after 1 day oral dosing in patients with Kellgren-Lawrence stage I, II or III osteoarthritis of the knee.

**Method:**

This randomized, double-blinded, 3-period crossover, phase II study compared 1 day (2 doses) of NEO6860 (500 mg twice a day), placebo, and naproxen in 54 patients with osteoarthritis knee pain. Primary endpoint was reduction in pain intensity (PI) on Numerical Rating Scale after exercise, using the staircase test, 8 hours after dose.

**Results:**

Level of PI, compared with baseline, was numerically lower during NEO6860 and naproxen periods vs placebo at 3 and 24 hours, but not at 8 hours after first dose. A statistically significant effect for naproxen and a trend for NEO6860 were observed at 3 and 24 hours. Least square means' (95% confidence interval) change in PI at 24 hours was −0.67 (−1.09 to −0.26), −0.97 (−1.39 to −0.55), −0.29 (−0.71 to 0.13) for NEO6860, naproxen, and placebo, respectively. NEO6860 exposure was ∼1.6 times higher compared with previous phase I. In this study, NEO6860 safety profile was less favorable than naproxen or placebo. Possibly NEO6860-related adverse events included: feel hot, headache, nausea, dizziness, fatigue, hypoaesthesia, and increased blood pressure.

**Conclusion:**

In this exploratory study, NEO6860 did not statistically significantly outperform placebo but showed an analgesic trend, without impacting body temperature and heat pain perception. Further studies are warranted to explore the potential of NEO6860 in other pain indications. We intent to optimize the dose and evaluate analgesic synergism with other mechanism.

## 1. Introduction

Nearly 27 million people in the United States suffer from osteoarthritis (OA),^32^ a musculoskeletal disorder involving degradation of joints. Pain is the most debilitating symptom.^15^

Current pharmacotherapy approaches for the management of patients with OA have significant limitations in terms of efficacy and safety.^3,8^ A recent meta-analysis evaluated the pain-relieving effects of commonly used pharmacological agents for OA pain of the knee.^5^ The systemic pharmacological interventions demonstrated limited effect sizes ranging from 0.18 for acetaminophen to 0.38 for naproxen on a 0 to 10 pain scale.^5^ In addition, significant risks were associated with the use of nonsteroid anti-inflammatory drugs (NSAIDs) (mainly cardiovascular and gastrointestinal) or opioids.^4,10,27^

The transient receptor potential vanilloid subtype 1 (TRPV1) is localized in human sensory nociceptors and in neuronal fibres that carry noxious information up through layer I to II in the dorsal horn of the spinal cord. When activated, TRPV1 depolarizes the nerve ending, which results in nerve impulses that are perceived as pain. Experimentally, antagonists to TRPV1 are analgesic in animal pain models.^17^ Several companies have tested TRPV1 antagonists in humans. To the best of our knowledge, phase II studies have been initiated for at least 7 programs (AZD1386/AZ, AMG 517/Amgen, SB705498/GSK, JNJ39439335 [mavatrep]/J&J, MK-2295/Merck, GRC 6211/Glenmark, and DWP05195/Deawoong). Unfortunately, apart from 2 studies conducted with AZD1386^23^ and mavatrep^24^ in patients with OA, most of the phase II studies have not been published. The development of AZD1386 was terminated after observation of elevated temperature and loss of heat pain perception, which may limit the doses to a subefficacious range. Thermal burns and hyperthermia have been reported with mavatrep.^24^ It seems clear that most of these programs have been terminated for safety concerns, mainly hyperthermia^16^ or impaired noxious heat sensation.^29^

NEO6860―a new chemical entity pharmacologically categorized as an antagonist of the cloned human TRPV1 receptor―has a unique in vitro profile, described as modality-selective TRPV1 antagonist: NEO6860 antagonizes the capsaicin activation of human TRPV1 (IC_50_ = 41.5 nM, with 5%–15% partial agonism on its own), but has little or no activity against activation by heat (no effect up to 10 μM) or low pH (IC_50_ = 211 nM, 36% Imax) (data on file). It has been hypothesized that the profile of NEO6860 may result in analgesia without the effects on body temperature or heat pain threshold, which have been reported in the clinic with TRPV1 antagonists lacking the modality-selectivity of NEO6860.

In a double-blind, placebo-controlled, first-in-man phase I study, which included 64 subjects, NEO6860 demonstrated good bioavailability after oral ingestion, target engagement, and pharmacodynamic (PD) effects, as measured by the level of pain and secondary hyperalgesia after intradermal injection of capsaicin in healthy young volunteers.^12^

Based on these findings, a proof-of-concept phase II trial was initiated with NEO6860 in OA, where 2 TRPV1 non–modality-selective antagonists have been successfully tested.^23,24^

## 2. Materials and methods

### 2.1. Participants

Adult outpatients with pain associated with OA of the knee were recruited from 4 sites in Quebec, Canada, located in Montreal (2 sites), Sherbrooke, and Quebec City.

To be eligible, patients must have had a body mass index between 18.0 and 35.0 kg/m^2^; a diagnosis of OA of the knee according to American College of Rheumatology guidelines (at least 3 of the following: patient age >50, morning stiffness <30 minutes, crepitus on active motion, bone tenderness, bone enlargement, or no palpable warmth of synovium); or grade I, II, or III Kellgren–Lawrence classification of an X-ray of the knee (performed 6 months or less before inclusion). Grade I patients were limited to a maximum of 50% of the total number of patients; a Numerical Rating Scale (NRS) score ≥4 (maximum 10) after staircase test (described below); a Western Ontario and McMaster Universities Osteoarthritis (WOMAC, 3.1 Index Likert, 24-hour recall questionnaire) pain subscale score ≥6 (maximum 20); and an *R*^2^ of the Focused Analgesia Selection Task (FAST)^31^ outcome value >0.65, indicating the patient's ability to report pain accurately (Most patients were recruited using the last 3 criteria, but some were included before final amendment using other cutoffs for pain intensity [PI], WOMAC, and FAST. In any case at least one pain assessment, PI or WOMAC, must have been above 40% of maximum value, and 0.65 was the lowest FAST cutoff value used during the study).

Patients were excluded from the study if they met any of the following criteria: any clinically significant disorder (including fibromyalgia and other painful disorders) that may have interfered with the primary objectives of the study; treatment in the previous 3 months with topical capsaicin or intra-articular corticosteroids; and a contraindication for the use of naproxen or acetaminophen.

### 2.2. Study design and procedures

The study was a randomized, double-blinded, placebo-controlled, 3-period crossover study, where each patient received alternately 1 day (2 doses) of NEO6860 (500 mg twice a day [bid]), placebo, or naproxen (500 mg bid) in a random sequence. The procedures were approved by a central ethics committee (IRB Services, Aurora, Ontario, Canada), and the study was conducted according to the principles of the Declaration of Helsinki as well as the ICH Guidelines for Good Clinical Practice. The study was registered with ClinicalTrials.gov under identifier number: NCT02712957.^25^ No important changes were made in the methods after the study was initiated, except for the definition of the level of pain and cutoff for FAST. The randomization list was provided by the Biostatistics Department at JSS Research (Montreal, Quebec, Canada) and sequences were communicated using Interactive Web Response System. Patients, investigators, pharmacists, and study staff were blinded to the randomization sequence.

#### 2.2.1. Overall study design

During the study, each subject was exposed to each of those 3 drug products. After the screening period, subjects were randomized to one of the 6 treatment sequences. During each of the 3 dosing periods, subjects were requested to participate in 2 clinic visits: one 13-hour residential visit, during which patients received a dose of study drug in the morning and a second dose 12 hours later; and a second visit the next day for assessments (no dosing). Two washout periods of 1 to 3 weeks separated the dosing periods. For a subpopulation at one site, the residential period was extended to approximately 24 hours to allow for the assessment of heat pain threshold and tolerance.

#### 2.2.2. Summary of procedures

Before each of 3 treatment periods, and for 24 hours after the first dose of study drug in each period, patients were required to: (1) discontinue analgesic therapy, except for short-acting opioids or NSAIDs, for 1 week or at least 5 half-lives (whichever was shorter); (2) discontinue short-acting opioids or NSAIDs for 24 hours or at least 5 half-lives (whichever was longer); and (3) refrain from use of rescue medication (acetaminophen) for 24 hours. For each dosing, to ensure blinding, fasted patients received an oral liquid suspension in a bottle (containing NEO6860 500 mg or its placebo) and one capsule (overencapsulated tablet of naproxen 500 mg or its placebo). The second dose of study drug was given 12 hours after the first dose. Before and after the first dose, patients underwent an abbreviated physical examination, vital signs, 12-lead electrocardiography (ECG), the staircase test, and provided a blood sample for clinical laboratory evaluations and pharmacokinetic (PK) assessment. Pain intensity (outside of exercise) was measured hourly from predose to 12 hours after first dose, and at 24 hours after dose. The 24-hour postdose evaluations included an abbreviated physical evaluation, measurement of weight, 12-lead ECG, clinical laboratory evaluations, as well as 2 questionnaires: WOMAC (3.1 Index Likert)^2^ and Patient's Global Impression of Change (PGIC). Then, patients were released for a washout period.

After the 3 treatment periods, subjects returned for a poststudy visit, 7 to 10 days after the last dose, for an abbreviated physical examination, vital sign and weight measurements, 12-Lead ECG, any occurrence of adverse events (AEs), change in concurrent medical conditions, use of an adjunctive therapy/procedure, intake of concomitant medication, pregnancy test, and safety laboratory samples. End-of-study procedures were also performed with patients who discontinued treatment prematurely.

#### 2.2.3. Focused Analgesia Selection Task procedure

The FAST (Analgesic Solutions, Natick, MA) has been developed to measure patients' pain-reporting skills. The procedure consists of a total of 49 stimuli: 7 different temperatures, between 43 and 51°C, each presented 7 times. Stimuli are presented in random order, and the subject is asked to rate the PI at the peak of each stimulus. Both the stimulus order and responses are recorded by the software. Accuracy of pain reporting was measured using the *R*^2^ of the correlation between pain reporting and temperature, with a minimum score of 0.65 indicating an acceptable pain-reporting capability.^31^

#### 2.2.4. Staircase test

The StEPP (Staircase-Evoked Pain Procedure, Analgesic Solutions) is a stress test, performance-based outcome measure developed to improve the sensitivity to detecting analgesic effects in clinical trials of knee OA. Subjects were instructed to define an “index knee” (most painful knee) for all assessments. The task consisted of stepping fully up onto an 8-inch (20-cm) high platform with one foot then the other foot and back down (alternating lead leg at each up/down cycle), for a total of 24 times. Pain intensity assessments were performed immediately before and after the exercise using a 0 to 10 NRS.^24^

#### 2.2.5. Heat pain assessment procedures

Heat pain assessment was performed in a subpopulation (patients included at Algorithme Pharma, one of the site) as described in the study by Brown et al.^12^ except for these 2 modifications: (1) the cutoff temperature for these procedures was 51°C instead of 53°C and (2) a newer generation of the computer-controlled thermal sensory analyzer device (Medoc Q-Sense, Ramat Yishai, Israel) was used.

### 2.3. Outcome measurements

#### 2.3.1. Primary efficacy outcome

The primary efficacy endpoint was the change in index knee PI on the 0 to 10 NRS after the staircase test, from baseline to 8 hours after first dose.

#### 2.3.2. Secondary efficacy outcomes

The change from baseline PI as measured by: (1) before and after the staircase test, 3, 8, and 24 hours after dose; (2) before and after the staircase test, averaging 3-, 8-, and 24-hours postdose values; and (3) averaging hourly PI values at rest.

The Sum of Pain Intensity Differences (SPID) is defined as the sum of (PI_i_ − PI_baseline_) × time (hours) elapsed since the previous measurement. Poststaircase test PI values were used to calculate SPID8 (2 values: 3- and 8-hour postdose) and SPID24 (3 values: 3-, 8-, and 24-hour postdose). Hourly PI values before staircase tests or outside of staircase tests were used to calculate SPID12 (11 values), summing up the PI difference up to 12 hours after dose.

The WOMAC 24-hour recall questionnaire^2^ is a self-administered questionnaire consisting of 24 items divided into 3 subscales: pain (5 questions), stiffness (2 questions), and physical function (17 questions). Each question has a scale 0 to 4, where higher scores represent higher symptom severity. The pain subscale has 5 items: during walking, using stairs, in bed, sitting or lying, and standing so that WOMAC pain subscale ranges from 0 to 20. As secondary endpoints, the mean change from baseline in WOMAC score was assessed for each subscale and for the full scale, at the screening visit and 24 hours after dose.

The PGIC^19^ was assessed 24 hours after dosing, before patients were discharged, using the following 7-point scale: (1) No change (or condition has gotten worse), (2) Almost the same, hardly any change at all, (3) A little better, but no noticeable change, (4) Somewhat better, but the change has not made any real difference, (5) Moderately better, and a slight but noticeable change, (6) Better and a definite improvement that has made a real and worthwhile difference, or (7) A great deal better and a considerable improvement that has made all the difference.

#### 2.3.3. Use of rescue medication

Acetaminophen (provided as 500 mg tablets, to be used up to 3 g/d) was the analgesic rescue medication. Patients were asked to refrain from taking the rescue medication 24 hours before first dosing and 12 hours after last dosing, for each of the 3 treatment periods. The endpoint was a yes/no to taking any rescue medication during the study.

#### 2.3.4. Safety outcomes

Safety outcomes included physical examinations, vital signs, AEs, concomitant medications, and laboratory results. Data were collected starting at the screening visit and at all visits after study entry. In addition, change in heat pain threshold and tolerance was measured on a subpopulation of 11 patients from one study site (Algorithme Pharma, Montreal, Quebec, Canada).

#### 2.3.5. Pharmacokinetic outcomes

Blood samples were taken predose, then at 2, 3, 8, and 24 hours after first dose for the measurement of plasma concentration of NEO6860 to derive PK outcomes: area under the concentration time curve: AUC_0–8_ (ng·h/mL), AUC_0–24_ (ng·h/mL), AUC_0–∞_ (ng·h/mL); maximum concentration in plasma: C_max_ (ng/mL); time to maximum concentration: t_max_ (h); terminal elimination half-life: t_½_ (h); apparent clearance: CL/F (L/h); apparent volume of distribution: V/F (L), and accumulation ratio: R.

### 2.4. Data analysis

#### 2.4.1. Sample size

For 80% power, a sample size of 42 evaluable patients was calculated as sufficient. The sample size calculations were based on the primary efficacy comparison being between NEO6860 and the placebo, where the expected difference between these 2 treatments was 1.0 point on the 0 to 10 NRS, with a null hypothesis value of 0.0. It was also assumed that NEO6860 would be equivalent (δ = 0) to naproxen. Derived from the literature,^14,18,30^ SDs for the mean differences were estimated at 2.0 and 1.0, for the comparison of NEO6860 with placebo and naproxen, respectively. There were no interim analyses to this study.

#### 2.4.2. Analysis population and statistical analyses

The modified intent-to-treat population, defined as all randomized subjects who had at least one available postdose PI value, was used for the primary analysis population. The safety population was defined as all randomized subjects who have taken at least one dose of study medication. The per protocol (PP) population consisted of all randomized subjects who completed the study without major protocol violations.

Primary and secondary efficacy endpoint analysis included mean, median, SD, and 95% confidence intervals. The Student *t* test for paired comparisons was used to test the statistical significance of the difference if the mean change follows the normal distribution, and the nonparametric Wilcoxon rank sum test was used if not normally distributed.

A secondary analysis of the primary efficacy outcome was performed using a mixed-effects analysis of covariance model, in which the patient was the random effect; treatment group, treatment period, and randomization sequence were the fixed effects; and baseline PI was a covariate. Primary and secondary analyses of the primary outcome were repeated for the PP population. A *P* value below 0.05 was used to indicate significance in all analyses.

Adverse events were coded using the Medical Dictionary for Regulatory Activities (MedDRA, Version 18.1). The total number of AEs, and the total number and proportion of patients experiencing at least one AE during the treatment period were summarized by body system and preferred term. Adverse events were also described according to intensity and causal relationship to the treatment.

Pharmacokinetic outcomes were described using the statistics geometric mean and geometric coefficient of variation (%). The relationship between exposure (AUC_0–8_) and response (change in PI from baseline to 8-hour postdose) was assessed graphically with a scatter plot; the correlation was described with the Spearman's correlation coefficient.

## 3. Results

### 3.1. Patients' disposition and baseline data

**Figure 1.**
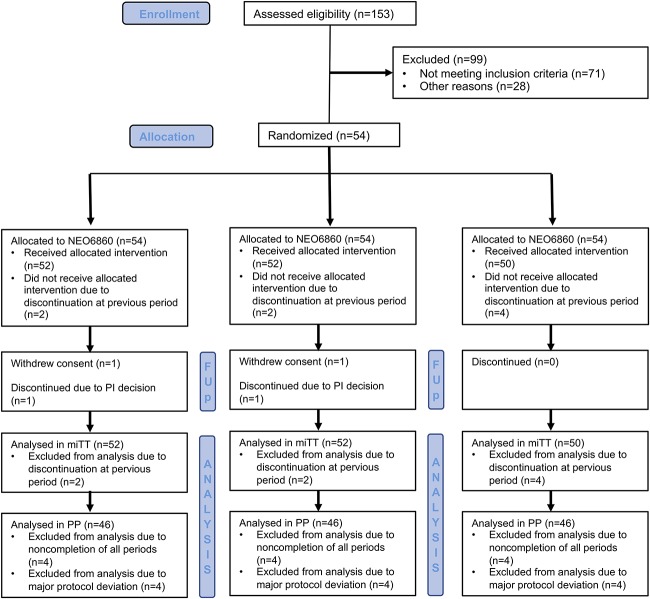
Participant flow diagram. Patients were actually randomized into 6 possible sequences of the 3 treatment modalities. “Allocated to” refers to the treatment period. FUP, follow-up patients; mITT, modified intent-to-treat; PI, principal investigator; PP, per protocol.

A total of 153 patients were screened and 54 patients were randomized (Fig. 1) between March and November 2016.

All randomized patients (n = 54) were included in the safety population (ie, have taken at least one dose). Eight patients were excluded from the PP analysis due either to noncompletion of all periods (n = 4; 7.4%) or to discontinuation (n = 4; 7.4%); hence, 46 patients were included in the PP population. Reasons for discontinuation were: consent withdrawn (n = 2; 3.7%) and principal investigator decision (n = 2; 3.7%).

The mean age of the 54 randomized patients was 61.1 years (SD = 9.06 years; range: 42.7–78.3 years), and a large proportion (87.0%) was older than 50 years (Table 1). All patients were Caucasian, and almost all were non-Hispanic (94.4%). The majority were female patients (63.0%). The median duration of OA was 5.2 years (range: 0.1–42.2 years). At baseline, a majority of the patients suffered from morning stiffness, crepitus on active motion, bone tenderness, and bone enlargement (Table 1).

**Table 1:**
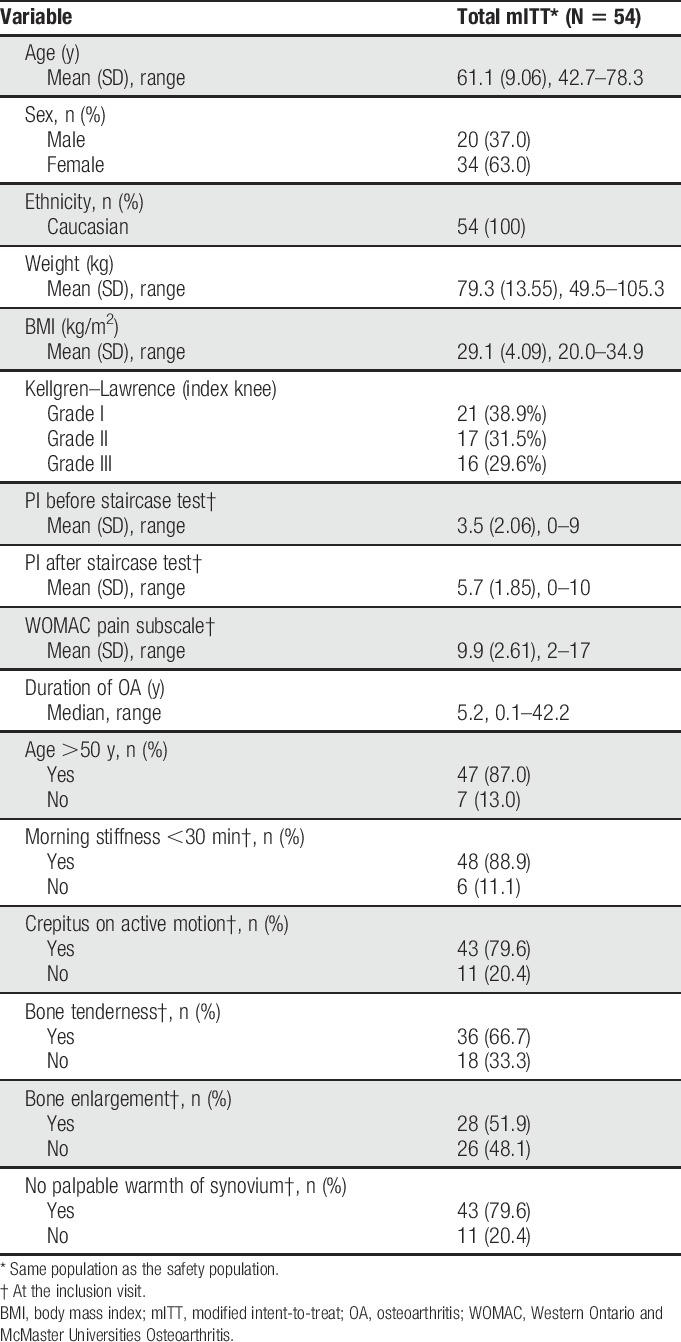
Patient demographics and history of OA of the knee at screening.

### 3.2. Primary endpoint

**Figure 2.**
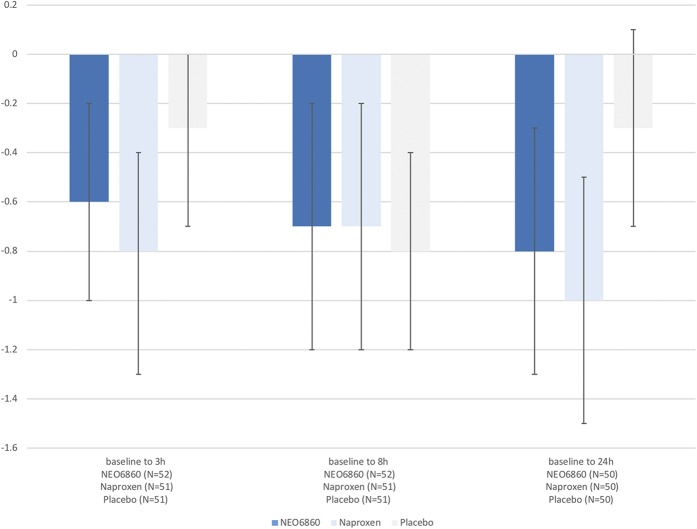
Absolute change in PI after the staircase test from baseline by treatment. Bars indicate 95% CIs in the mITT population. Mean (SD) PI values at baseline were 4.6 (2.06), 4.1 (2.09), and 4.2 (2.05) for NEO6860, naproxen, and placebo, respectively. CIs, confidence intervals; mITT, modified intent-to-treat.

The mean change (SD) in PI from baseline after the staircase test to 8-hour postdose was −0.7 (1.66), −0.7 (1.72), and −0.8 (1.39), respectively, for NEO6860, naproxen, and placebo. No significant difference was demonstrated between NEO6860 and placebo (*P* = 0.746) or between naproxen and placebo (*P* = 0.457) (Fig. 2).

### 3.3. Secondary endpoints

#### 3.3.1. Poststaircase pain

The changes in PI from baseline to 3 hours or 24 hours, measured after the staircase test, revealed a reduction in the level of pain for both NEO6860 and naproxen compared with placebo (Fig. 2). This difference was statistically significant at the 24-hour time point for naproxen vs placebo (*P* = 0.046). For NEO6860 vs placebo, the *P* value was 0.19 at the 24-hour time point (Table 2).

**Table 2:**
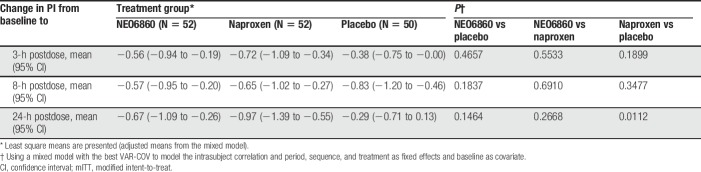
Absolute change in PI after the staircase test from baseline using the mixed model in the mITT population.

Results of the general mixed analysis of covariance model, controlling for period, sequence, and treatment as fixed effects and baseline by period (time-dependent) as covariates, are presented in Table 2.

Using a mixed-effects repeated-measures general linear model with respect to poststaircase pain, no period or sequence effect was detected.

Before the staircase test, ie, no exercise/rest pain, the absolute change in PI values for NEO6860 and naproxen was numerically superior to that of the placebo, although not significantly different (*P* ≥ 0.05), as shown in Table 3. Mean (SD) baseline values were 3.2 (2.38), 2.7 (1.81), and 2.6 (2.14) for NEO6860, naproxen, and placebo, respectively.

**Table 3:**
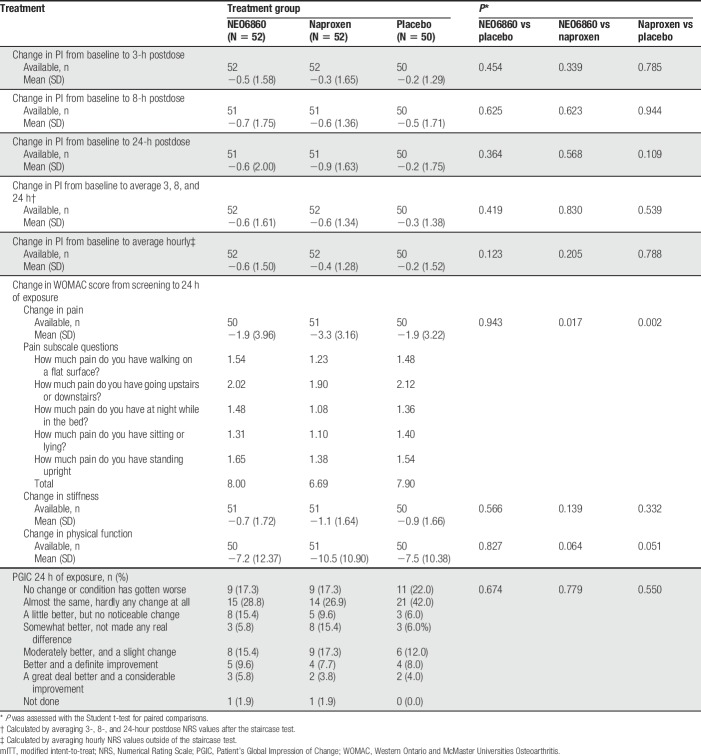
Rest pain, WOMAC, and PGIC assessment in the mITT population.

#### 3.3.2. Sum of Pain Intensity Differences

Pain intensity results were reflected in the SPID8, SPID24 (using poststaircase NRS values), and SPID12 (using NRS at rest), where a trend was also observed, but no significant difference was detected between NEO6860 and the placebo, or between NEO6860 and naproxen (*P* > 0.05) (data not shown). The mean (SD) SPID24 was −17.4 (39.62) for NEO6860 and −9.7 (29.09) for placebo (*P* = 0.280). The mean (SD) SPID12 for NEO6860 and placebo was −6.0 (17.27) and −2.6 (17.77), respectively (*P* = 0.208).

#### 3.3.3. Western Ontario and McMaster Universities Osteoarthritis

The mean change from baseline in WOMAC pain subscale score was not significantly different between NEO6860 and placebo, although an effect was detected for naproxen (Table 3).

#### 3.3.4. Patient's Global Impression of Change

**Figure 3.**
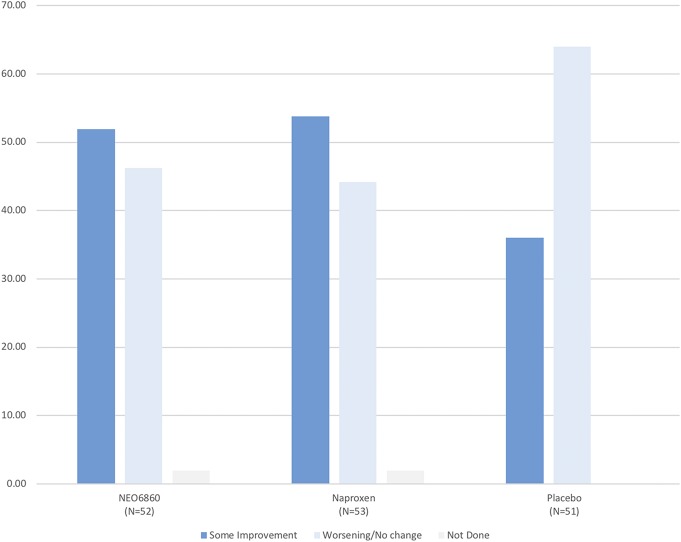
Patient's Global Impression of Change according to 2 categories in the mITT population. *P*-values were 0.0736 for NEO6860 vs placebo and 0.0201 for naproxen vs placebo using the Row Mean Score test (controlling for within-subject correlation). mITT, modified intent-to-treat.

The majority of patients in each group assessed their impression of change after 24 hours of exposure as “almost the same, hardly any change at all” (28.8% for NEO6860; 26.9% for naproxen; and 42.0% for placebo), which was slightly more in the placebo group; however, no significant difference was detected between NEO6860 and placebo or naproxen for the PGIC (*P* = 0.674 and *P* = 0.779, respectively) (Table 3). When grouped into 2 categories (“some improvement” vs “worsening or no change”), more patients reported “some improvement” with NEO6860 and naproxen when compared with placebo (Fig. 3). Differences were statistically significant when comparing naproxen with placebo (*P* = 0.0201) and close to significance when comparing NEO6860 with placebo (*P* = 0.0736).

#### 3.3.5. Use of rescue medication

Approximately 90% of patients in each treatment group took acetaminophen, the allowed rescue medication during the study (*P* = 0.960), which happened during the washout period and never during the treatment period. The median number of tablets taken during the study varied from 17 to 22 by treatment group (SD ∼20 tablets) (data not shown).

#### 3.3.6. Safety outcome measures

The majority of reported AEs were mild (94.2%, 61.4%, and 52.0%, respectively, for the NEO6860, naproxen, and placebo groups) (Table 4). “Feeling hot” was the most commonly experienced AE possibly related to the treatment, which was mainly reported by patients in the NEO6860 group (90.4%, 1.9%, and 8.0% of patients for the NEO6860, naproxen, and placebo groups, respectively) (Table 5). Other commonly reported AEs possibly related to the treatment include: headache, nausea, dizziness, fatigue, increased blood pressure/hypertension, and hypoaesthesia. Severe events of feeling hot and headache were reported each by one patient (1.9%) from the NEO6860 group. Both events were resolved.

**Table 4:**
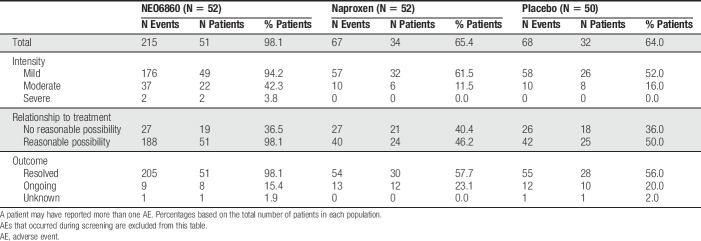
Overview of AEs in the safety population.

**Table 5:**
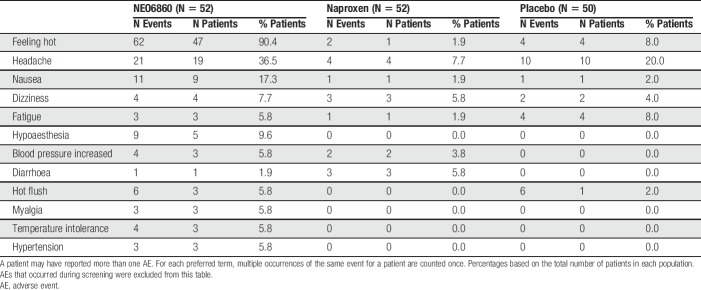
Most commonly reported (≥5% of patients) AEs possibly related to study treatment in the safety population.

Events of feeling hot were experienced mostly after the first administration of study drug rather than after the second administration (45/52 [87%] patients vs 2/52 [4%] patients in the NEO6860 group, 2/52 [4%] patients vs 0/52 [0%] patients in the naproxen group, and 2/50 [4%] vs 1/50 [2%] patient in the placebo group, for the first and second administration, respectively).

As per vital sign assessments, oral body temperature was monitored. At no time during the study was an increase in temperature of 1°C or more reported (Table 6), including the patient who reported a severe sensation of feeling hot. No clinically significant abnormalities were reported after other vital signs' measurements, laboratory evaluations, or ECG evaluations.

**Table 6:**
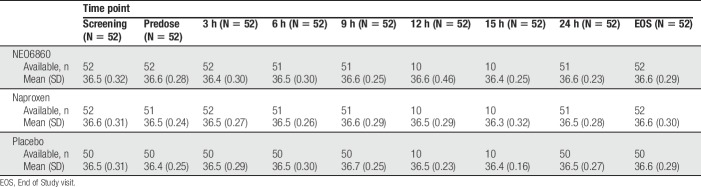
Oral body temperature per time point and treatment modality in the safety population.

#### 3.3.7. Heat pain threshold/tolerance

Conducted on a subpopulation from one study site, the assessment of change in heat pain threshold and heat pain tolerance did not show noticeable difference between treatments (NEO6860 and placebo) or between pretreatment and posttreatment (Table 7).

**Table 7:**
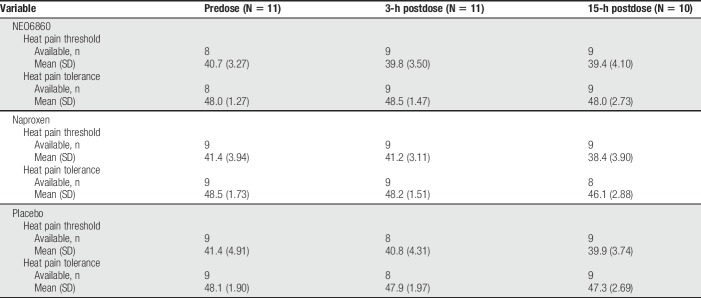
Heat pain threshold and tolerance in a subset of patients (°C).

### 3.4. Pharmacokinetic outcome measures

NEO6860 maximum concentration in plasma (geometric mean C_max_ = 4336.61 ng/mL, CV = 32.5%) was reached in 3.60 hours (t_max_; CV = 44.8%). Other parameters (ie, t_1/2_, CL/F, V/F, and accumulation ratio, *R*) are not reported because the number of time points did not allow for a reasonable assessment. NEO6860 mean (±SD) plasma concentrations were 3304 (±1419), 4287 (±1370), 3100 (±1248), and 2702 (±1420) ng/mL respectively, 2, 3, 8 and 24 hours after the first dosing.

#### 3.4.1. Pharmacokinetic/pharmacodynamic relationships

An exposure-response scatter plot was built with exposure (as measured by AUC_0–8h_) on one axis and response (as measured by change in PI from baseline to 8 hours after dose) on the other axis. No correlation was observed (Spearman's coefficient at −0.037, data not shown).

## 4. Discussion

Patients reported a reduction in pain after the staircase test with NEO6860 and naproxen, at 3, 8, and 24 hours after dose, although the difference was statistically significant only for naproxen vs placebo (*P* = 0.046) at the 24-hour time point. At 24-hour postdose, the treatment effect observed with naproxen vs placebo was 0.7 points. This finding is in line with data reported in a recent meta-analysis,^5^ validating our model at this particular time point.

The 8-hours time point was selected as the primary outcome measure because a medically and commercially viable compound needs to be administered a maximum of 3 times daily (eg, every 8 hours). The primary endpoint (8-hour change from baseline in PI after staircase) was not statistically significant for naproxen or NEO6860. Although the change from baseline effects of naproxen and NEO6860 were comparable at all time points, the placebo effect was larger at the 8-hour time point than at the 3- and 24-hour time points. This situation could be because the 8-hour time point came after a long rest for all patients because they remained relatively inactive during the treatment period in the unit. This might have limited the capacity to see a treatment effect, whereas at 24 hours, conditions were similar to the ones at baseline for most patients, ie, coming from home in the morning. Alternatively, pain in patients with OA undergoes either a linear increase or a U-shaped diurnal variation, with PI in the first pattern low in the morning then gradually increasing until the evening, or in the second pattern high in the early morning, quickly decreasing, then gradually increasing during late afternoon and evening.^6^ This variation in PI may at least partially explain the variation in placebo effect. In any case, the lack of difference between the active control arm, naproxen, and placebo invalidates the 8-hour time point.

Based on the change in PI after staircase test at the 24-hour time point, NEO6860 induced a reduction in PI of 0.5 points compared with placebo. Using Hedges g effect sizes, as in the meta-analysis by Bannuru et al.,^5^ NEO6860 effect is comparable or slightly below the reported analgesic effect of celecoxib (Hedges *g* at 0.30 and 0.33 for NEO6860 and celecoxib in the meta-analysis, respectively) and above that of acetaminophen (0.18).

Results of the WOMAC questionnaire, comparing naproxen with placebo, also helped validate the study design, showing a treatment effect similar to the one reported by Bannuru et al.^5^ No difference was observed between NEO6860 and placebo in the WOMAC questionnaire, but heterogeneity was observed in the pain subscale. For instance, the second question (How much pain do you have going upstairs or downstairs?) revealed the highest level of pain during the placebo period (2.12 of 4 points) while pain levels were 2.02 and 1.90 for NEO6860 and naproxen, respectively, reproducing the pattern observed with PI after staircase test. We and others have observed unexpected behavior of the WOMAC pain subscale in TRPV1 studies: a phase II study assessing AZD1386, another TRPV1 antagonist, showed that despite a clear treatment effect (vs placebo) based on change in PI (*P* = 0.0087), there was no significant effect on WOMAC (*P* = 0.3086).^23^ Similarly, mavatrep demonstrated a greater analgesic effect using PI than with WOMAC.^24^ Of note, in the same publication,^24^ at the 24-hour time point, mavatrep and naproxen were not different from placebo on the WOMAC pain subscale, suggesting that this scale may not be optimal after only 1-day dosing. On the contrary, the PGIC questionnaire yielded similar effect for NEO6860 and naproxen, which was more favorable than that of the placebo group.

The high plasma exposure observed in this population was surprising. At 500 mg bid, the C_max_ was 4337 ng/mL, ie, ∼1.6 times higher than in the first-in-man phase I study at the same dose (Of note, at the highest dose levels in the phase I study, 1200 mg, the C_max_ was 3600 ng/mL). This dosage was selected for the phase II study because in phase I, it was well tolerated, had an optimal PD effect, and one day-2 doses' PK data were available. This finding is not fully understood because no patient had liver insufficiency in the phase II study population (NEO6860 is metabolized by the liver). Nevertheless, we believe that the exposure measured in these 52 patients with OA is more relevant than previous data established in a limited number of healthy volunteers (N = 6 for each dose group).

Importantly, and despite the high exposure, none of the historical TRPV1 AEs (hyperthermia^9,16,33^ and impaired noxious heat sensation^11,20,21^) were observed in the NEO6860 group. It should be noted that with non–modality-selective TRPV1 antagonists, these 2 AEs were dose-dependent and reported in most (and sometimes in all) subjects at high exposure levels so that lack of such observations with NEO6860 is likely not attributable to the limited sample size of our studies (phase I and phase II). Similarly to that reported in the first-in-man phase I study,^12^ most of the AEs reported in this study were mild in intensity. Headache, nausea, dizziness, fatigue, and hypoaesthesia were the most commonly reported AEs. Although 90% of patients in the NEO6860 group reported a sensation of feeling hot, its incidence decreased drastically between the first administration and the second administration (from 87% to 4%). Such a decrease was also reported in the first-in-man phase I study.^12^ Feeling hot has been previously reported with TRPV1 antagonists,^24^ albeit not at such frequency. No mouth-burning sensation was reported, although NEO6860 was administered as a liquid oral form. The underlying mechanism for this feeling hot sensation is not fully understood and deserves further exploration. This event was responsible for the most significant limitation of our trial: feeling hot was indicative of NEO6860 treatment allocation. We believe that the bias was limited by the 3-modalities design, where absence of this event could be associated with either no activity (placebo period) or established active analgesic (naproxen period).

In this proof-of-concept study, NEO6860 was associated with more AEs than naproxen and placebo. The very high exposure generated by the 500-mg bid dosing likely explains the suboptimal safety profile observed in this study. As observed in preclinical studies and in the phase I study,^12^ it is expected that the frequency and severity of AEs will decrease with reduced dosing.

The hypothesis that motivated this program is that blocking the capsaicin activation of the TRPV1 channel, while not inhibiting its heat- or pH-induced activation, would result in a compound that would trigger analgesia without inducing high body temperature and impairment of heat pain perception. Here, we confirm initial observations in the phase I study^12^ demonstrating that NEO6860 does not produce elevation of core body temperature nor impacts high temperature perception. However, although an analgesic trend is observed, this study failed to reach the primary endpoint at 8 hours. The potential analgesic properties of NEO6860 should be further studied in additional clinical trials.

In the phase I study, the maximum PD effect (on evoked pain and secondary hyperalgesia) was not observed at the highest level of exposure, suggesting a nonlinear PK/PD relationship. Therefore, the NEO6860 dose could potentially be significantly reduced, perhaps by up to 10-fold, without negatively impacting its efficacy.^12,13^ This is corroborated by an absence of correlation between exposure (as measured by AUC_0–8h_) and PI reduction in the present phase II study, suggesting that patients were already at the plateau of efficacy. Pain intensity after staircase test increased steadily from 3 to 24 hours, suggesting that NEO6860 potential analgesic effect could be time-dependent and that analgesia could be improved with multiple dosing.

The safety of high-dose NSAIDs is a growing concern with a demonstrated increased risk of major cardiovascular events.^3,7,26,27^ We and others have demonstrated in animal models using isobolographic measurements that the combination of a TRPV1 antagonist with various NSAIDs results in a synergistic increase in analgesia^22,28^ (and data on file). Thus, a combination of NEO6860 (dose to be determined) with low-dose NSAIDs may prove to be more effective than high-dose NSAIDs, potentially making NEO6860 an improvement in the current paradigm of analgesic therapies.

In addition, the number of deaths due to opioid overdoses in the United States is a major concern: according to the Center for Disease Control, in 2016, there were 32,445 deaths involving prescription opioids,^1^ reinforcing the need for new, safe, effective, nonopioid medications for chronic pain.

## 5. Conclusion

In this limited exploratory proof-of-concept study, NEO6860 did not demonstrate superiority to placebo but showed an analgesic trend without impacting body temperature and heat pain perception. The analgesic trend of NEO6860 is in the range of celecoxib and below that of naproxen. The safety profile of NEO6860 was suboptimal, likely due to unexpectedly high levels of NEO6860 exposure.

Future investigations should focus on exploring the potential of the unique pharmacological profile of NEO6860 in various pain indications (neuropathic pain, pain associated with chronic pancreatitis, etc.) while reducing the exposure of NEO6860 and improving the safety profile. Furthermore, NEO6860 should be tested in OA, in combination with low-dose NSAIDs, to improve analgesia and potentially alleviate safety concerns of high-dose NSAIDs.

## Disclosures

The authors have no conflict of interest to declare.

This study was sponsored by NEOMED Institute. The authors are employees of NEOMED Institute (D.C., W.B., and P.W.) or paid consultants of NEOMED Institute (J.M., R.L., R.T., and N.K.).
